# Training on domestic violence and child safeguarding in general practice: a mixed method evaluation of a pilot intervention

**DOI:** 10.1186/s12875-017-0603-7

**Published:** 2017-03-04

**Authors:** Natalia V. Lewis, Cath Larkins, Nicky Stanley, Eszter Szilassy, William Turner, Jessica Drinkwater, Gene S. Feder

**Affiliations:** 1 Centre for Academic Primary Care, School of Social and Community Medicine, University of Bristol, Canynge Hall, 39 Whatley Road, Bristol, BS8 2PS UK; 2 School of Social Work, Care and Community, University of Central Lancashire, Harrington Building, Preston, PR1 2HE UK; 3 School for Policy Studies, University of Bristol, Social Science Complex, 8 Priory Road, Bristol, BS8 1TZ UK; 4 Leeds Institute of Health Sciences, Charles Thackrah Building, 101 Clarendon Road, Leeds, LS2 9LJ UK

**Keywords:** General practice, Family practice, Domestic violence, Child safeguarding, Child protection, Pilot projects, Evaluation report, Pre-post-tests, Qualitative evaluation, Quantitative evaluation

## Abstract

**Background:**

Children’s exposure to domestic violence is a type of child maltreatment, yet many general practice clinicians remain uncertain of their child safeguarding responsibilities in the context of domestic violence. We developed an evidence-based pilot training on domestic violence and child safeguarding for general practice teams. The aim of this study was to test and evaluate its feasibility, acceptability and the direction of change in short-term outcome measures.

**Methods:**

We used a mixed method design which included a pre-post questionnaire survey, qualitative analysis of free-text comments, training observations, and post-training interviews with trainers and participants. The questionnaire survey used a validated scale to measure participants’ knowledge, confidence/ self-efficacy, and beliefs/ attitudes towards domestic violence and child safeguarding in the context of domestic violence.

**Results:**

Eleven UK general practices were recruited (response rate 55%) and 88 clinicians attended the pilot training. Thirty-seven participants (42%) completed all pre-post questionnaires and nine were interviewed. All training sessions were observed. All six trainers were interviewed. General practice clinicians valued the training materials and teaching styles, opportunities for reflection and delivery by local trainers from both health and children’s social services. The training elicited positive changes in total outcome score and knowledge and confidence/ self-efficacy sub scores which remained at 3-month follow up. However, the mean sub score of beliefs and attitudes did not change and the qualitative results were mixed. Two interviewees described changes in their clinical practice. Participants’ suggestions for improving the training included incorporating more ethnic and class diversity in the material, using cases with multiple socio economic disadvantages, and addressing multi-agency collaboration in the context of changing and under-resourced services for children.

**Conclusions:**

The pilot training for general practice on child safeguarding in the context of domestic violence was feasible and acceptable. It elicited positive changes in clinicians’ knowledge and confidence/ self-esteem. The extent to which clinical behaviour changed is unclear, but there are indications of changes in practice by some clinicians. The pilot training requires further refinement and evaluation before implementation.

**Electronic supplementary material:**

The online version of this article (doi:10.1186/s12875-017-0603-7) contains supplementary material, which is available to authorized users.

## Background

Although the negative health impact of domestic violence and abuse (DVA) is well documented [1], training for health care professionals on how to identify and respond to patients experiencing DVA is virtually absent from undergraduate and postgraduate curricula [2, 3] and has a patchy presence within the continuing professional education [4–6]. A lack of special training is one of the multiple barriers prevailing clinicians from asking patients about DVA and adequately responding to disclosure [7–9]. DVA and child safeguarding (CS) are inter-related issues because (i) direct exposure to DVA has a negative impact on children’s health and well-being to the extent that it can be considered maltreatment [10] and (ii) there is an overlap between direct child maltreatment and DVA [11–15]. The strong connection between DVA and child maltreatment is recognised in UK national guidance [16]. Health care professionals’ key role in CS has been highlighted in recent policy developments [17, 18]. Opposite to DVA, training on CS for general practice is well established [16], but the link to DVA is rarely made [19]. General practitioners (GPs) are reported to remain uncertain of their CS responsibilities in the context of DVA [20]. Training to identify and manage DVA and, where appropriate, refer children affected by DVA to children’s services is therefore required [21].

The pilot training evaluation reported here was a component of the RESPONDS study (*R*esearching *E*ducation to *S*trengthen *P*rimary care *on D*omestic Violence and *S*afeguarding) which integrated several evidence sources into the development of a training on DVA and CS for general practice [20, 22–26]. The aim of this study was to pilot the training and evaluate its acceptability, feasibility and the direction of change in short-term outcome measures. We hypothesised that the RESPONDS training would: (i) be acceptable to general practice clinicians and feasible to deliver; (ii) increase clinicians’ knowledge on DVA and CS policy and procedures; (iii) increase clinicians’ confidence and self-efficacy in supporting families exposed to DVA; (iv) improve clinicians’ attitudes towards women and children exposed to DVA; (v) encourage reflection on clinicians’ own practice regarding DVA and CS.

## Methods

### Design

We used a mixed method design [27–29] which included a pre-post questionnaire survey and qualitative analysis of free text comments from the survey, training observations, and post-training interviews. Based on previous research [30, 31], we expected low response rates from general practice staff and decided to use multiple data sources to obtain the maximum amount of data. Mixed methods enabled us to explore: (i) context for training and the experiences of trainers and participants (free text comments, observations, interviews); (ii) the extent to which learning is put into practice by participants (interviews, survey); (iii) the direction of changes in knowledge, confidence, attitudes and clinical behaviour (interviews, survey). Qualitative data helped contextualise and interpret the quantitative findings by enabling us to explore factors which may have facilitated or limited change in individual or collective practices [32].

We applied investigator triangulation, drawing on researchers from different academic disciplines, as well as methodological triangulation in order to ‘cancel out’ and double check any convergence, inconsistency, and contradiction that may arise within one paradigm. The aim of this was to increase confidence in the findings [27, 33]. Triangulation was both concurrent and sequential [34]. This enabled development from one element of the study to another [27]. Interim analysis of findings from the survey and the concurrent training observations informed the development of interview schedules for trainers and participants [35].

### Study procedures

The Primary Care Research Network assisted with identification, sampling and recruitment of general practices for this study. We chose two geographical areas in south England and the Midlands, and drew a purposive sample of general practices of different size, location, ethnicity, socio-economic status, experience of previous DVA training, and provision of local DVA services. To increase practice response rate, we have followed strategies recommended for research in general practice [36]. Thus, each participating practice received a £500 incentive. All training participants received Continuing Professional Development (CPD) certificates stating that the RESPONDS session generated two hours of CPD in accordance with the current Royal College for General Practitioners (RCGP) Appraisal and Revalidation Guidance [37].

One week before the training (T1), practice administrators e-mailed all practice staff an invitation to the study and a link to an on-line SurveyMonkey questionnaire [38]. On the day of the training, researcher observing the session requested that all attendees complete the questionnaire if they had not already done so and provided a laptop for this task. Practice administrators emailed a second invitation and the survey link immediately after the training (T2). A third invitation was e-mailed three months post-training (T3). To increase response rate, weekly reminders were emailed at each time point. In order to match the pre- and post-training responses while protecting anonymity, the participants created a unique identification code which they used at the three time points. All the training sessions were observed. Telephone and face-to-face interviews were carried out with trainers at T2 and with training participants at T3.

After the training, we asked general practice teams to review their recording policies on DVA and CS. If they did not have recording policies, we requested to develop these in light of the received training. We asked practices to return the revised documentation to the research team within a month following the training.

The study was guided by two panels of professionals and service users who contributed to developing the interview schedule and analysis.

### Intervention

The training aimed to fill gaps in knowledge and practice on the interface between DVA and CS revealed by the RESPONDS research [20, 22]. Development of the training is reported in detail elsewhere [26]. In brief, the training pack was developed collaboratively using clinical, academic, front line practitioner, service user and training expertise in DVA, CS and health. The RESPONDS training was focused on: (i) needs of children exposed to DVA emphasizing the safety of children and their non-abusive parents, (ii) management of adults and children living with DVA in the same family, (iii) the importance of consistent responses from general practice, DVA and children’s social services. The content of the training addressed the following issues: (i) linking DVA and CS in practice; (ii) holding difficult conversations about DVA and speaking directly with children; (iii) responding to DVA disclosure; (iv) child protection referral process; (v) working with other professionals; (v) record keeping, safety and confidentiality.

An overarching feature of the RESPONDS training was to teach how to link DVA and CS in practice [39]. Multi-agency work with organisations who commission and provide services for children and families was emphasised throughout the training. The training focused on how clinicians can work with other professionals in supporting patients with experience of DVA while negotiating a child protection referral to ensure the safety of their children. Training provided information on how to seek advice and work with other health care professionals and third sector DVA agencies; it explained the role of children’s social services, and described what general practice clinicians can expect following a child protection referral and what referral information is most helpful to provide. It also discussed the importance of inter-agency work in providing support for both threshold and sub-threshold children and their families. The training also taught how clinicians can provide support to perpetrators.

Each training session was delivered by two trainers, a health care professional and a local children’s social work professional. To ensure fidelity to the model, all trainers attended the ‘train the trainer event’. The training was delivered to individual general practice teams as a 2-h safeguarding level 3 [40] session during lunchtimes on each practice premises. Informed by the review of previous effective training in this field [22] as well as by the qualitative interviews and consultations with DVA experts and survivors [26], the teaching was interactive with an emphasis on discussion and reflection on practice. The session incorporated a film in which a female patient presented to a GP with depression and described her concerns about the behaviour of her teenage son. The GP asked the mother about DVA, spoke individually to the patient’s son to elicit his experiences, and then discussed with the mother next steps, including making a referral to children’s social service. The film was interspersed with short narratives from practicing GP and a social worker. These experts provided guidance on overcoming challenges faced in general practice when dealing with DVA and CS.

### Quantitative measures and statistical analysis

The on-line questionnaire included a demographic section and validated outcome measure. The demographic section requested information about gender, age, job position and job experience. Participants were also asked whether they had a designated safeguarding role and whether they previously received DVA training. Ample free text space was provided at the end of the questionnaire to enable participants to comment on the training and to write down their contact details if they were interested in taking part in follow up interviews. Survey responses were downloaded from the Survey Monkey software [38] and imported into Stata [41] for subsequent analysis.

The validated outcome measure was the modified Domestic Abuse and Safeguarding Children Scale (M-DASC). An additional text file shows the M-DASC in detail [see Additional file 1]. The original DASC was developed for the evaluation of the inter-professional training in DVA and CS from a number of items used in previous research [42]. A group of six DVA and CS experts selected 10 items from the original DASC that were applicable to the general practice context and added 17 new items on identification and response to women and children exposed to DVA. The M-DASC consisted of 27 items with responses endorsed on a 1-5 scale (1 =’Strongly disagree’ to 5 = ‘Strongly agree’); possible range of total score was 1 to 135. Working through group discussion and consensus, the experts designated three M-DASC subscales: (1) knowledge of DVA and CS (16 items, possible range of scores 1-80), (2) confidence/ self-efficacy in responding to women and children exposed to DVA (14 items, range 1-70), and (3) beliefs/attitudes towards DVA (8 items, range 1-40). Some items appeared in more than one subscale [see Additional file 1]. We used total score and three subscale scores as continuous measures for the quantitative analysis.

To assess test-retest reliability of the M-DASC, a Pearson's product-moment correlation was used to measure the relationship between the two sets of repeated questions [43]. Internal consistency of the M-DASC was assessed with a Cronbach’s alpha [44]. Frequencies were used to present categorical variables. We used the Fisher’s exact test to compare two proportions for small samples [45].

We hypothesised that there would be positive changes in M-DASC measures taken post-training compared to the pre-training estimates, and used repeated-measures ANOVA to test the hypothesis [45]. The primary outcome was total M-DASC score; the secondary outcomes were three M-DASC sub scores. Time was a within-subject variable with three categories (T1, T2, T3). Between-subject covariates (gender, age, job position, job experience, previous DVA training) were pre-specified based on published research [46, 47]. We first ran a main analysis on a sample of participants who provided data at all three time points and then carried out a sensitivity analysis on a sample of participants who provided data at least twice (including at T1) . The results of the two analyses were then compared. We present a complete case analysis.

#### Test-retest reliability and internal consistency of the modified Domestic Abuse and Safeguarding Childrenscale

We assessed test-retest reliability of the M-DASC with 29 social work students at the University of Bristol. The students completed the questionnaire twice with a 2-week gap. Total scores and sub scores at both time points were highly correlated: (i) total score (r (27) = 0.79; *P* < 0.001); (ii) knowledge sub score (r (27) = 0.76; *P* < 0.001); (iii) confidence/self-efficacy sub score (r (27) = 0.74; *P* < 0.001); (iv) beliefs/ attitudes sub score (r (27) = 0.69; *P* < 0.001).

The Cronbach alpha (α) for the whole M-DASC had a very good level of internal consistency: 0.86 at the first time point and 0.91 at the second time point. [48]. Two subscales also demonstrated good levels of internal consistency at both time points (knowledge α = 0.82 and 0.86 and confidence/ self-efficacy α = 0.79 and 0.88, respectively). However, the beliefs/attitudes subscale had internal consistency below minimally acceptable level at the two time points (α = 0.22 and 0.52, respectively).

### Qualitative data and analysis

The qualitative element of the study had inductive and hypothetical logic. Working inductively from our earlier research [22, 42, 49] and existing literature [46, 50–52] we theorised that the process (delivery of and engagement with trusted training materials, group participation, and co-delivery by trainers from health and social care) would provide opportunities for reflection and that would subsequently lead to outcomes (increased knowledge of policy and procedures, increased confidence and self-efficacy, improved attitudes towards DVA and CS, and reflection on own role and practice). This informed our training observation guide [see Additional file 2] and interview schedule for trainers [see Additional file 3], which focused on documenting successes and challenges in the process and exploring initial outcomes (moments where training participants appeared to exhibit changes). We created an interview schedule for training participants [see Additional file 4] based on these observations, trainer interviews and the survey results at T2. This enabled us to explore concerns about factors which appeared to facilitate or limit the intervention delivery and where M-DASC measures indicated no change. Interviews were conducted by telephone or face-to-face, audio recorded, transcribed verbatim, and coded in NVivo [53].

The data were analysed using content analysis against our inductively theorised process and outcome measures (as above) to identify individual, local and system factors relevant to practices towards DVA and CS [27, 54]. From our previous research [42, 49] and literature [46, 50–52] we knew that gender, practice culture, and the availability of time and information about local resources were likely to be relevant to an individual’s practice. We also speculated that individual pre-training attitudes and availability of support during the training were likely to be relevant to the direction of change in outcomes. Content was coded based on major themes identified in our previous research [26, 42, 49]. Each interview and free text comment was analysed systematically and coded according to the existing thematic coding structure. Additional codes were added where new themes emerged from the data and previous interviews and free-text comments were re-read and re coded with new codes if relevant.

Participants’ quotes from survey free text comments are reported as ‘Survey GPID’, from observations as ‘ObsID’, from interviews with trainers and clinicians as ‘TrID’ and ‘TGPID’, respectively.

## Results

### Sample characteristics

Invitation letters and study information sheets were sent to 10 practices in the South and 10 practices in the Midlands. Overall, seven southern and five Midlands practices agreed to participate. However, one southern practice withdrew due to unexpected doctor capacity issue which resulted in 11/20 practices enrolled in the study (55% response rate). All southern practices had previous DVA training, although not all clinicians had been exposed to the training. The previous DVA training was received through IRIS – an evidence-based training, support and referral programme for general practice [4]. None of the Midland practices received previous DVA training.

Between May and July 2014, we delivered pilot training to 88 general practice clinicians. One pair of trainers worked with Midlands practices, the other two pairs worked with southern practices. The training team comprised of three health care professionals (named GP for safeguarding children, designated nurse for child protection, and liaison nurse from MARAC (Multi-Agency Risk Assessment Conference)) and three social workers (Early Help co-ordinator, children's social care senior practitioner, independent social worker). All the training sessions were observed by one of three researchers. All six (female) trainers were interviewed post-training (T2).

Overall, 25 training participants (21 in the South and four in the Midlands) expressed their interest in taking part in the follow up interviews (T3). However, four participants moved practice or went on maternity or long term sick leave, and seven did not reply to our email or phone invitations. In addition, five participants withdrew from follow up interviews due to increased workload. Consequently, we interviewed nine training participants (seven in the South and two in the Midlands; seven female, two male; two South Asian, seven white; all GPs; one aged 25-34; two aged 35-44; two aged 45-54; two aged 55-65; two safeguarding leads).

In total, 82/88 (93%) training participants completed the questionnaire at T1, 73/82 (60%) completed post-training survey at T2, and 42/82 (34%) completed 3-month follow up at T3. One questionnaire had all items missing and was excluded from analysis at this stage. After matching the questionnaires by ID, 37 participants had survey data at all three time points, 27 at two time points, and 18 at one time point. We coded those who completed all three questionnaires as ‘completers’ and those who did not provide data at all three time points as ‘dropouts’. Data from survey completers who comprised 42% (37/88) of those trained were used in main statistical analysis. We then used 63/88 cases with at least two valid responses including T1 (72% of those trained) in a sensitivity analysis.

For 32 of the 42 variables used in this analysis, less than 10% of values were missing. Ages of the participants at T1 ranged between 25 and 64 years [Table 1]. All age groups were equally represented. Over three-quarters were GPs, followed by practice nurses and ‘other professionals’ (two practice managers, one health care assistant, one phlebotomist, one pharmacist). Nearly equal proportions of the participants had been practising for up to 20 years and for more than 20 years. About 20% of participants (16/77) had a designated safeguarding role. About 39% of respondents (30/77) received DVA training through IRIS [4]. When we compared survey completers and dropouts by their characteristics at T1, we found evidence of a selective dropout. Those participants who provided data at all three time points, and were thus included in the complete case analysis, were more likely to be IRIS-trained and to work in the South.

**Table 1:** Comparison of survey completers and dropouts on socio-demographic and background characteristics

Variable	Categories	Total *n* = 82	Dropouts *n* = 45	Completers *n* = 37	*P*
		*n* (%)	*n* (%)	*n* (%)	
Gender	Female	55 (67)	27 (60)	28 (76)	0.16
	Male	27 (33)	18 (40)	9 (24)	
Age	<25-34	16 (20)	12 (27)	4 (11)	0.09
	35-44	23 (28)	13 (29)	10 (27)	
	45-54	25 (30)	9 (20)	16 (43)	
	55-64	18 (22)	11 (24)	7 (19)	
Job title	GP	63 (77)	34 (76)	29 (78)	0.52
	Nurse	14 (17)	8 (18)	6 (16)	
	Admin/ manager	2 (2)	2 (4)	0 (0)	
	Other	3 (4)	1 (2)	2 (5)	
Years of practice	0-9	20 (24)	15 (33)	5 (13)	0.08
	10-20	24 (29)	13 (29)	11 (30)	
	>21	38 (47)	17 (38)	21 (57)	
Safeguarding role	No	61 (79)	33 (79)	28 (80)	1.00
	Yes	16 (21)	9 (21)	7 (20)	
IRIS trained	No	47 (61)	38 (90)	9 (26)	<0.001
	Yes	30 (39)	4 (10)	26 (74)	
Geographic area	South	54 (66)	18 (40)	36 (97)	<0.001
	Midlands	28 (34)	27 (60)	1 (3)	

*Note:* Proportions are reported for available data. *IRIS* Identification and Referral to Improve Safety training, *SD* standard deviation, *P* p-value for the Fisher’s exact test

### Qualitative evaluation of training acceptability and feasibility

#### Engaging and trustworthy training materials and delivery style

Participants opinions about training materials and delivery style were collected to evaluate the acceptability and feasibility of the training through exploring: (i) whether participants thought appropriate and trustworthy information was delivered (interviews); (ii) how they engaged with the video (interviews, observations); (iii) how well they thought the training had been delivered (interviews, observations); (iv) whether the training was compatible with participants’ existing knowledge (survey, interviews, observations). We also assessed delivery style (observation, interviews) and trainers’ experiences of delivering the training and engaging participants (observations, interviews).

Training participants considered that appropriate information was well presented and thoroughly delivered. Trainers were described as ‘non-threatening’ (TGP02) and the materials were compatible with participants’ existing knowledge.

Training participants were observed to engage well with the video; it was ‘realistic, very good’ (TGP03) and ‘very powerful’ (TGP07) because it involved:

*…seeing the GP actually talk to the child and all the different stages, and then discussing it, that was really useful, very different from just talking about it” (TGP07).*

Two respondents did not ‘remember the video too well’ (TGP02) or the child (TGP01) but saw the video as making them ‘more alert’ (TGP01) or provoking ‘thoughts and ideas’ (TGP02). One respondent who thought the video was unnatural and unrealistic saw the video as a prompt for team discussion on approaches that ‘we all felt were more appropriate’ (TGP09). In observations and trainer interviews, the suggested broad filter questions to patients experiencing DVA, were particularly valued with some saying ‘I will definitely use that’ (Obs04) or ‘that's a good thing to ask’ (Tr01). However occasional resistance to engagement was observed, particularly in three practices described by one trainer as ‘old school’ (Tr02). One GP responded to the mother in the video by saying ‘well if she will nag’ (Obs1).

Suggested improvements included videos that were more ‘concise and punchy’ (TGP07), ‘multi-ethnic’ (TGP02), ‘class diverse’ (TGP03) or ‘a bit more complicated’ (TGP08). Some participants felt that the training should address drug and alcohol use, parental non-consent and how to deal with ‘an incredibly dysfunctional under-resourced market of referral services’ (TGP05).

#### Opportunities for reflection

All interviewed participants concurred that there were ‘enough’ or ‘definitely enough’ opportunities for reflection, and seeing ‘how my colleagues deal with it’ (TGP01) was perceived as particularly useful. However we observed, and all interviewees agreed, that the extent to which training delivery enabled reflection on participants’ own clinical cases depended on their experience. Two respondents suggested that this reflection case work could be a follow up training event. However, 10 of the 11 practices did not complete the post-training review of their recording policies on DVA and CS.

#### Group engagement

Group engagement was assessed through exploring how participants engaged in the group work (interviews, observations). The interviewees thought that most of their colleagues engaged in group discussions; it was ‘authoritative but low key … sort of join, join in and everybody felt very safe’ (TGP04) and ‘one of the best things’ (TGP09). But in some practices, we observed a marked lack of engagement in group discussions, as on one occasion trainers felt ‘we were just talking into an empty space’ (Tr03) or groups were dominated by ‘one [usually senior] doctor who would do a lot of the talking’ (Tr01). The trainers agreed that talking a lot could mean they ‘had a lot of questions and a lot to say’ (Tr03), whereas ‘not talking’ did not mean ‘not participating’ (Tr02). One trainer suggested facilitating greater parity of engagement or participation through ‘small groups, all at a similar [knowledge] level’ (Tr02).

#### Provision of local multi-agency information

Each observed training session was delivered by health and social care frontline workers. Trainers reported the importance of multiagency delivery and this was echoed by participants:

*“I think it's absolutely fantastic having professionals who are dealing with this day and day out” (TGP06).*

The ‘social services input was that extra thing’ (TGP08) that took training delivery beyond that delivered through IRIS which had no input from social worker. The RESPONDS training was ‘able to defend that [social services] message rather than somebody who is just [a trainer]’ (TGP05).

### Quantitative analysis of training outcomes

In the questionnaire survey completed by the training participants at T1, M-DASC demonstrated a very good level of reliability of the whole scale (α = 0.91) and two subscales (knowledge subscale α = 0.85; confidence/self-efficacy subscale α = 0.88). However, the beliefs/attitudes subscale had reliability below minimally acceptable level (α = 0.51) [48].

The training elicited positive changes in total M-DASC score and two sub scores which remained at 3-month follow up [Table 2].

**Table 2:** Modified Domestic Abuse and Safeguarding Children Scale (M-DASC) scores before and after training intervention (*n* = 37)

Scale/ subscale score	T1		T2		T3		*P*
	Mean	SD	Mean	SD	Mean	SD	
Total	84.4	10.6	96.1	7.1	95.2	8.7	<0.001
Knowledge	50.5	7.0	59.1	5.0	58.2	5.4	<0.001
Confidence/self-efficacy	42.8	6.3	49.5	4.5	49.1	5.8	<0.001
Beliefs/attitudes	24.5	1.8	24.8	1.9	24.7	2.0	0.61

*Note*: T1 – pre-training. T2 – immediately post-training. T3 – 3-month follow up. *SD* standard deviation, *P p*-value for the repeated-measures ANOVA test

Compared with T1, the mean total M-DASC score increased by 11.7 units at T2 and stayed 10.8 units higher three months later (F (2, 108) = 19.83, *P* < 0.001). Compared with T1, the mean knowledge sub score increased by 8.6 units at T2 and remained 7.7 units higher at T3 (F (2, 108) = 24.02, *P* < 0.001). The confidence/self-esteem sub score was 6.7 and 6.3 units higher than at T1, respectively F (2, 108) = 16.72, *P* < 0.001).

The mean measure of beliefs and attitudes did not change either post-training (increase by 0.3 units) or at follow up (increase by 0.2 units) (F (2, 108) = 0.23, *P* = 0.61). It is worth noting that at the start of the training, scores on five out of eight items on this subscale [see M-DASC items 3, 5, 7, 9, 20 in Additional file 1] already indicated strong beliefs and positive attitudes towards DVA and CS which remained unchanged throughout the study (mean scores of 4 out of 5 possible).

**Fig. 1:**
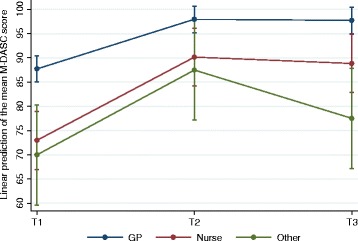
Adjusted predictions of the effect of job position and RESPONDS training on total M-DASC score. Note. GP – general practitioners. Nurse – practice nurses. Other – other primary care practitioners. T1 – at the start of the RESPONDS training. T2 – immediately after the RESPONDS training. T3 – 3-month follow up. Line shows mean total M-DASC scores and 95% confidence intervals at three time points

We found some evidence that mean M-DASC score of GPs was higher compared to other general practice clinicians both at the start of the training and at 3-month follow up [Fig. 1]. At T1, mean M-DASC score of GPs was 87.7 (95% CI 85.0 to 90.4), compared to 73.0 of nurses (95% CI 67.0 to 79.0) and 70.0 of ‘other practitioners’ (95% CI 59.7 to 80.3). At T2, all professional groups improved their scores and the gap disappeared (97.9 vs. 90.2 and 87.5, respectively, all three 95% CIs overlap). At T3, although the total effect of the training remained, mean M-DASC score of GPs stayed higher (97.7, 95% CI 95.0 to 100.4) compared to nurses (88.8, 95% CI 82.9 to 94.8) or ‘other professionals’ (77.5, 95% CI 67.2 to 87.8).

**Fig. 2:**
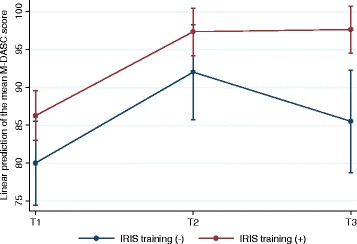
Adjusted predictions of the effect of IRIS training and RESPONDS training on total M-DASC score. Note. IRIS – Identification and Response to Improve Safety – domestic violence training, support and referral programme for general practice. IRIS training (-) – participants without previous IRIS training. IRIS training (+) – participants with previous IRIS training. T1 – at the start of the RESPONDS training. T2 – immediately after the RESPONDS training. T3 – 3-month follow up. Line shows mean total M-DASC scores and 95% confidence intervals at three time points

We found some evidence of an interaction between previous IRIS training and the RESPONDS training, indicating that IRIS was associated with more sustainable M-DASC score between T2 and T3 (F (2, 58) = 3.81, *P* = 0.05) as seen from the plateau effect in Fig. 2 between T2 and T3. There was no difference in the M-DASC score by previous IRIS training either at T1 (mean difference between two categories 6.3, 95% CI -0.2 to 12.7; *P* = 0.06) or at T2 (mean difference 5.3, 95% CI -1.7 to 12.3; *P* = 0.14). However, at 3-month follow up, mean M-DASC score of IRIS trained respondents stayed 12.1 units higher compared to their non-IRIS trained colleagues whose mean score dropped (95% CI 4.7 to 19.6; *P* = 0.02).

Sensitivity analysis performed on a sample of participants who had valid responses at least at two time points including T1 (*n* = 63) produced results similar to the analysis of 37 study completers who had valid responses at all three time points.

### Qualitative evaluation of training impact on individual and collective practices

#### Knowledge

Most qualitative interviews supported the survey finding of increased knowledge post-training. All eight interviewees described some new or ‘refreshed’ (TGP06) knowledge. This included ‘quite powerful learning about the impacts on children of domestic violence’ (TGP04), and that ‘people are actually very happy to just have the questions asked’ (TGP03). Knowing ‘the next steps’ was seen to enable ‘better conversation with the mother” (TGP06) and had led this GP to decide:

*“The next time that I do [a referral], that's what I'm going to do…because if we're going to be all proactive, we want to make sure that we're involved in the loop” (TGP03).*

For IRIS-trained participants, learning about appropriate DVA recording was, ‘[not] anything we hadn't already heard’ (TGP05). However it was striking that in three non-IRIS practices the content on the use of codes in record-keeping appeared to be new, especially for junior colleagues (Obs. 2, 4 & 5), and one of those practices went on to develop a new recording system (TGP02).

Current information about ‘where to send them for help’ (TGP01) (e.g., a website for young people, MARAC, the Early Help Scheme) was valued in both IRIS trained and non-IRIS practices. Even those who knew all the agencies mentioned nonetheless felt the training enabled them to ‘know who to speak to’ (TGP07) or it provided ‘a lot more of a framework in my thinking’ (TGP03) on how to access support. Understanding children’s social services was particularly important. Respondents reported ‘an understanding of where [the referral] goes’ (TGP04). One GP had chosen to refer a DVA case to social services despite earlier negative experiences of no action following a referral. Another participant noted that in the future she would:

*“more readily [make] a referral to Social Services for, you know, a sort of a supportive basis, than I would have done previously… [training was useful] in terms of perhaps lowering the threshold when I might talk to them” (TGP09).*

However, one survey respondent ‘actually felt more muddled after this training’ because ‘there does seem to be an impressive array of disjointed services available.’ (Survey GPb). One example of the increased knowledge of referral mechanisms was seen to emerge at the local level, as Trainer 2 noted that ‘one of the practices had [subsequent to the training] made a referral, so something had obviously gone in’.

#### Confidence and self-efficacy

In line with the survey results, six interviewees explained that their ‘general improved confidence’ (TGP05) was ‘sort of built up’ (TN2) through being given information about role expectations, ‘owning the subject’ (TGP04), and developing understanding of how to proceed in consultations:

*“[The training] made me feel particularly confident about discussing this with children, you know, being able to ask them how it was affecting them …being able to raise that with the mum …. It has changed my management a bit of a couple of patients I've seen since” (TGP07).*

However, confidence remained ‘still not very high’ where ‘the child is … indirectly affected [by DVA]’(TGP01), where the survey respondent had not had time to apply the learning in practice (Survey GPa) or because they had increased awareness of ‘the reality of the limited service on offer by social services to support children if there is not consent.’ (Survey GPc).

#### Beliefs and attitudes

In contrast to the survey result of no change in the mean measure of beliefs and attitudes, qualitative findings were rather mixed. Thus, participants in two sessions were observed (Obs 2 & 5) to make the link from DVA to CS when they had not done so before or ended up ‘agreeing with the whole idea that you need to …treat it as a safeguarding issue’ (Tr05). However, Trainer 4 remained concerned that training participants in other sessions ‘were still speaking of them as two separate issues’. Five interview respondents identified changes in their attitudes regarding the potential effects of children’s exposure to DVA and the need to ask children about DVA, contrasting this to their previous approach:

*“If I saw children with disturbed behaviour I tended not to think, you know, could it be due to difficulties at home? … And I think that [training] completely changed my mind, so I actually always ask that now every time I see a child with behaviour problems” (TGP07).*

Although some training participants ‘looked quite shocked that the GP in the video suggested talking to the child,’ encouragement to talk to a child alone came through discussion with colleagues (Tr03).

For those who remained hesitant about talking to children, this was because they ‘possibly would not look for the child’ if the mother had not brought the child in (TGP01); they felt ‘unclear [what] my role is in terms of actually bringing the child in for a separate consultation’ (TGP05); or, were ‘generally much happier to talk to children’ but still reluctant ‘to muddy the waters’ by talking to a child directly in a specific case (TGP03).

In three training sessions, the researchers observed and trainers identified having ‘not enough of a focus on making the link from DVA to children’ (Tr04) or the need to feel ‘a bit more confident in making such link’ (Tr02). One GP with experience in a paediatric post and a safeguard lead explained that his attitudes towards abused women were already positive before the training.

#### Engagement in reflection on own clinical practice

Only one out of eleven practice carried out the follow up review of their DVA and CS recording policies. Engagement in reflective practice was not addressed by an M-DASC subscale and there is no evidence of change on the one statement most directly indicative of reflective practice ‘I understand how my own experiences may influence my capacity and willingness to engage with issues of domestic violence and abuse’ [see M-DASC item 3 in Additional file 1]. Changes in reflective practice were not mentioned by the training participants we interviewed. When asked directly, one participant said the practice was already ‘quite keen on training and then encouraging reflective practice’ (TGP02) and a second said that training had provided a rare opportunity for reflection.

## Discussion

General practice clinicians and trainers found the RESPONDS training in DVA and CS acceptable and feasible. They valued the engaging and trustworthy materials and teaching styles, opportunities for reflection and delivery by trainers from local health and children’s social services. The questionnaire survey showed that the training increased clinicians’ knowledge and confidence/ self-efficacy, but did not change their beliefs/attitudes towards DVA and CS. This could be because the participants’ responses at the start of the training already showed that they understood women experiencing abuse and were positive about engaging with issues of DVA. Perhaps those practitioners who underwent previous DVA training were more familiar with these issues or their positive attitudes were unrelated to the DVA training.

Qualitative findings were also mixed. Some clinicians were more confident in knowing how to proceed in a consultation, and had greater awareness of relevant service provision and referral routes. Some participants reported increased willingness to engage directly with children and to discuss this with the non-abusive parent and this led to some changes in case management. Some participants from practices without previous DVA training learned about recording and were developing new systems. However, some participants reported no change in their clinical behaviour and some felt more confused after the training.

Participants’ suggestions for improving the training included incorporating more ethnic and social class diversity in the material, using cases with multiple deprivation and socio economic disadvantages, and addressing multi-agency collaboration in the context of changing and under-resourced referral services.

Engaging primary care clinicians in face-to-face training proved challenging and the planned follow-up review of recording policies on DVA and CS was not carried out by most practices. Low follow up rates in the survey and interviews indicate the difficulty of engaging general practice clinicians beyond training. The fact that only 55% of approached practices took part in the RESPONDS training suggests that there is a systematic barrier to upskilling general practice clinicians. Echoing Sahin and colleagues [55], the most cited reasons for non-participation in the training and evaluation were clinicians’ need to prioritise clinical care over research and lack of protected time.

The differential follow up rates between the southern IRIS trained practices and Midland’s non-trained practices, to both the survey and interview requests, could indicate a possible positive effect of previous IRIS training on engaging primary care clinicians with this public health and clinical problem. The southern practices were more likely to take part in the evaluation, suggesting some of the arguments about putting time aside for this issue had been won. It could be that IRIS trained participants were more likely to engage because they were familiar with the topic and with the DVA clinical champion involved in the RESPONDS video. The extent to which the selective follow up rate was associated with practice characteristics (e.g., clinician-patient ratio) is not known as we did not collect such data. The low response to the follow up review of recording policies on DVA and CS by IRIS trained practices could be explained by the fact that they already had recording policies as a result of previous training which was highlighted in the qualitative evaluation. This demonstrates the variability of DVA management and support in general practice across the UK, and the challenges of producing national training material, with activities suitable to all levels of experience.

Our findings are partly consistent with the systematic review of educational interventions on responses to children exposed to DVA [22]. This showed that training interventions improved participants’ self-reported knowledge, attitudes and clinical competence up to a year post intervention. Our results showed that those training participants who completed the study already had positive attitudes about DVA and CS at the start of the training. This could be explained by the IRIS training which most RESPONDS completers received in the past. Such previous DVA training and on-going practice support through IRIS programme could generate further interest and self-motivation among clinicians to further develop their knowledge, confidence/ self-esteem and skills and to participate in research about DVA.

This study found few examples of changes in clinical behaviour after the RESPONDS training. Interviews suggested behaviour change in some individuals such as increased willingness to ask about DVA and talk to children. However, other interviewees reported no change in their clinical behaviour. There are many possible explanations for this. For example, practitioners could be convinced, but did not have resources to change their own practice, or they could make changes but did not report back to the researchers. Another explanation might be the clinicians’ negative previous experience liaising with children’s social services. This demonstrates how changing clinical behaviour may require more time for reflection and reinforcement, perhaps longer training with booster sessions, as well as protected time and on-going support for implementation. The systematic review of DVA training interventions found evidence of the effectiveness of whole systems approaches; this might involve combining training for health care practitioners with wider system level interventions, such as increasing awareness of DVA, tools for victim identification, and improved access to support services [56].

### Strengths and limitations

This study evaluated an evidence based pilot training on DVA and CS using a mixed method approach which provided insight into training feasibility, acceptability and short-term outcomes. The design included a reliable measure of outcomes, the M-DASC scale and pre/post evaluation at three time points. Running the pilot in two sites allowed the identification of the value of previous DVA training and on-going support through the IRIS programme. The qualitative data provided possible explanations of why change did and did not occur, giving valuable guidance for future refinement of the RESPONDS training.

There are a number of shortcomings to this evaluation that limit its external validity. The first weakness is the absence of a control group, therefore we cannot be certain that changes in clinicians’ outcomes were due to the RESPONDS training rather than other factors. The second limitation is a measurement bias. The questionnaire survey relied on self-report, which might be hindered by social desirability and memory biases with a potential to exaggerate the effect of the training. The M-DASC had limited validation before use in this study and demonstrated poor internal consistency on the beliefs/ attitudes sub scale. Training observations were taken by one of three researchers. The third limitation is a selective bias. Poor engagement of general practice clinicians resulted in small sample size for qualitative interviews. As a result of selective dropout, we ended up with the sample of survey completers biased towards clinicians who had previously received IRIS training and were safeguarding leads, although our follow up rates (60% at T2 and 34% at T3) were higher than those reported in similar studies [42, 57]. The selective bias is particularly problematic in assessing changes in beliefs and attitudes where it is possible that only those clinicians who had positive attitudes towards DVA and CS participated in the evaluation of the RESPONDS training.

### Further research

Given the mixed results of the RESPONDS pilot, we propose further refinement and testing of the training. The further refinement could include an amendment of the teaching material with regards to diversity. Further evaluation should explore clinical impact such as changes in the rate of patient disclosure of past or current DVA, and potential harms in the form of parental anxiety, child fear or anxiety and inappropriate referral. Such evaluation requires a controlled study design, adequate sample size and long-term follow up.

As a stand-alone intervention the RESPONDS training could be implemented after refinement, but there remains uncertainty about its effectiveness in actually changing clinician behaviour, improving outcomes for families experiencing DVA and its potential for integration with other DVA training for general practice. Ultimately we do not think that RESPONDS should be a stand-alone training intervention, but should be integrated into other DVA interventions for general practices, combining it with training about the identification of and response to female and male patients with experience of DVA. For women patients, we already have IRIS – a training, support and referral programme. IRIS was tested in a cluster randomised trial and demonstrated clinical effectiveness [4], cost effectiveness [58] and acceptability to patients [59] and clinicians [60]. Following the success of the trial, IRIS has been implemented in >30 localities in England and Wales [61]. For male patients who have experienced or perpetrated DVA, we have a pilot study of HERMES, a training and support programme modelled on IRIS which showed that there was an increase in the identification and documentation of male patients following the pilot training [62].

As a result of the positive findings of the IRIS trial and promising results of the HERMES and RESPONDS pilots as well as responding to the needs of clinicians preferring the DVA training to be connected and have simpler referral routes, we aim to combine these three interventions into an integrated programme (IRIS+) and test its effectiveness in a cluster randomised controlled trial, powered to detect difference in identification and appropriate referral of men, women and children exposed to DVA. We will test whether the IRIS+ model, which builds on IRIS, but includes elements of the HERMES and RESPONDS is practical and whether it is equally effective at identifying female patients, as well as male patients experiencing/perpetrating DVA and their children [63].

### Policy and practice implications

A priority for policy and guidance is training on DVA and CS that addresses positive practice aimed at ensuring the safety of children and their parents exposed to DVA, with content regarding appropriate management of adults and children living in the same family [21]. The development of the RESPONDS pilot training and some promising findings of this evaluation are important steps in addressing those priorities in the general practice.

## Conclusions

The pilot RESPONDS training in DVA and CS for general practice was feasible and acceptable to both trainers and participants. It had a positive effect on clinicians’ knowledge and confidence/ self-esteem. The extent to which beliefs/ attitudes and clinical behaviour changed is unclear. However there are indications of changes in practice for some individuals. The training package requires further refinement and further, more rigorous evaluation before implementation.

## Additional files

Additional file 1: Modified Domestic Abuse and Safeguarding Children Scale items before and after training intervention (*n* = 37). Note. T1 – pre-training. T2 – immediately post-training. T3 – 3-month follow up. DVA – domestic violence and abuse. Reverse score items are 4, 5, 7, 8, 12. SD – standard deviation. (DOCX 15 kb)
Additional file 2: Training observation framework. (DOCX 16 kb)
Additional file 3: Training evaluation interview schedule – trainers. (DOCX 17 kb)
Additional file 4: Training evaluation interview schedule – training participants. (DOCX 17 kb)

## Abbreviations

ANOVA: Analysis of variance
CS: Child safeguarding
DASC: Domestic Abuse and Safeguarding Children scale
DVA: Domestic violence and abuse
GPs: General practitioners
ID: Unique identification number
IRIS: Identification and Referral to Improve Safety
MARAC: Multi Agency Risk Assessment Conference
M-DASC: Modified Domestic Abuse and Safeguarding Children Scale
RESPONDS: *R*esearching *E*ducation to *S*trengthen *P*rimary *c*are *on D*omestic violence and *S*afeguarding
