# Supplementary material for: Training on domestic violence and child safeguarding in general practice: a mixed method evaluation of a pilot intervention
Source: BMC Fam Pract. 2017 Mar 4;18:33. doi: 10.1186/s12875-017-0603-7 (PMC5336644; doi:10.1186/s12875-017-0603-7)
Supplement: Additional file 1: — Modified Domestic Abuse and Safeguarding Children Scale items before and after training intervention (n = 37). Note. T1 – pre-training. T2 – immediately post-training. T3 – 3-month follow up. DVA – domestic violence and abuse. Reverse score items are 4, 5, 7, 8, 12. SD – standard deviation. (DOCX 15 kb) [file 12875_2017_603_MOESM1_ESM.docx]

| **Scale item**  ***Sub scale*** | **T1** | | **T2** | | **T3** | |
| --- | --- | --- | --- | --- | --- | --- |
|  | **Mean** | **SD** | **Mean** | **SD** | **Mean** | **SD** |
| 1. I feel comfortable asking patients about DVA *Confidence/ self-efficacy* | 3.7 | 1.0 | 4.0 | 0.4 | 4.0 | 0.5 |
| 2. I would personally feel confident that I could correctly identify a woman with experience of DVA  *Confidence/ self-efficacy* | 3.1 | 0.7 | 3.5 | 0.6 | 3.5 | 0.6 |
| 3. I understand how my own experiences may influence my capacity and willingness to engage with issues of DVA  *Knowledge*  *Beliefs and attitudes* | 3.8 | 0.8 | 4.0 | 0.6 | 4.1 | 0.4 |
| 4. If I was to ask every woman who I suspect may be experiencing DVA if she has been abused, I will offend a lot of my patients  *Confidence/ self-efficacy*  *Beliefs and attitudes* | 2.1 | 0.6 | 1.9 | 0.5 | 1.9 | 0.5 |
| 5. Abused women should leave their partners, whatever the circumstances  *Beliefs and attitudes* | 2.2 | 0.7 | 2.0 | 0.7 | 2.1 | 0.7 |
| 6. Children witnessing incidents of DVA are at great risk of significant harm  *Knowledge* | 4.4 | 0.6 | 4.2 | 0.9 | 4.5 | 0.8 |
| 7. It is not my place to interfere with how a couple chooses to resolve conflicts  *Beliefs and attitudes* | 2.2 | 0.8 | 2.2 | 0.8 | 2.1 | 0.7 |
| 8. A woman should expect to be re-abused if she decides not to take appropriate action after being offered help/advice  *Knowledge*  *Beliefs and attitudes* | 2.2 | 1.0 | 2.3 | 1.0 | 2.0 | 1.0 |
| 9. It is my responsibility to ask a woman patient if she is experiencing DVA, given appropriate indication  *Knowledge*  *Beliefs and attitudes* | 4.4 | 0.6 | 4.4 | 0.6 | 4.4 | 0.5 |
| 10. I feel equipped with strategies to help ‘victims’ of DVA change their situation  *Confidence/ self-efficacy* | 3.5 | 1.0 | 4.0 | 0.7 | 3.9 | 0.8 |
| 11. I would know what to do if a parent disclosed DVA to me  *Knowledge*  *Confidence/ self-efficacy* | 3.9 | 0.7 | 4.3 | 0.5 | 4.2 | 0.5 |
| 12. Primary clinicians should only ask a woman about DVA if they strongly suspect she has experience of domestic violence and abuse  *Knowledge*  *Confidence/ self-efficacy* | 2.3 | 0.9 | 2.1 | 0.7 | 2.1 | 0.8 |
| 13. I feel confident in being able to ensure the safety of children while actively supporting the victim and maintaining an ongoing relationship with the family  *Knowledge*  *Confidence/ self-efficacy* | 2.9 | 0.9 | 3.5 | 0.7 | 3.6 | 0.8 |
| 14. I know how to actively support children and families who live with DVA but at the present time do not reach the threshold for a child protection service  *Knowledge* | 2.5 | 1.0 | 3.6 | 0.7 | 3.6 | 0.7 |
| 15. I know when and how to raise the issue of DVA with children  *Knowledge* | 2.6 | 0.8 | 3.7 | 0.6 | 3.6 | 0.6 |
| 16. I feel confident in talking directly to children about their experiences of DVA  *Confidence/ self-efficacy* | 2.4 | 0.7 | 3.4 | 0.7 | 3.5 | 0.7 |
| 17. I know how and when it is appropriate to talk to perpetrators about DVA  *Knowledge*  *Confidence/ self-efficacy* | 2.5 | 0.9 | 3.3 | 0.7 | 3.4 | 0.7 |
| 18. I have a good understanding of local information sharing policies for DVA  *Knowledge* | 3.2 | 0.9 | 4.1 | 0.6 | 4.0 | 0.4 |
| 19. I feel confident that I can make an appropriate referral for children of abused patients  *Confidence/ self-efficacy* | 3.5 | 1.0 | 4.0 | 0.7 | 3.9 | 0.7 |
| 20. Primary care clinicians should be more involved in identifying DVA cases  *Beliefs and attitudes* | 4.0 | 0.6 | 4.1 | 0.5 | 4.1 | 0.6 |
| 21. I am comfortable discussing safety issues/plans with abused patients  *Confidence/ self-efficacy* | 3.6 | 0.8 | 4.0 | 0.5 | 3.9 | 0.5 |
| 22. Even in ten minutes I can provide help and support for victims of DVA  *Confidence/ self-efficacy*  *Beliefs and attitudes* | 3.5 | 1.0 | 3.9 | 0.8 | 4.0 | 0.7 |
| 23. I know when, how and where to safely record disclosure and suspicions of DVA  *Knowledge* | 3.4 | 0.8 | 3.9 | 0.5 | 3.9 | 0.6 |
| 24. I can identify the different risks for different family members in families where there is DVA  *Knowledge*  *Confidence/ self-efficacy* | 2.9 | 0.7 | 3.8 | 0.6 | 3.6 | 0.7 |
| 25. I know how to act in order to increase the safety of all family members involved in DVA  *Knowledge* | 2.9 | 0.6 | 4.0 | 0.4 | 3.8 | 0.6 |
| 26. I know how to contact local DVA services and what help they can offer children and families  *Knowledge* | 3.7 | 0.9 | 4.2 | 0.4 | 4.1 | 0.6 |
| 27. I can explain to patients what they can expect from children’s social services following a referral  *Knowledge*  *Confidence/ self-efficacy* | 2.9 | 0.9 | 3.8 | 0.5 | 3.8 | 0.8 |
