# Supplementary material for: Training on domestic violence and child safeguarding in general practice: a mixed method evaluation of a pilot intervention
Source: BMC Fam Pract. 2017 Mar 4;18:33. doi: 10.1186/s12875-017-0603-7 (PMC5336644; doi:10.1186/s12875-017-0603-7)
Supplement: Additional file 2: — Training observation framework. (DOCX 16 kb) [file 12875_2017_603_MOESM2_ESM.docx]

Date: Environment: _____________

**Actors in the room:** Nos. participants (roles): Safeguarding lead present?

Ethnicity: Gender: Age estimates:

**Context**

Local issues/specificities/concerns?

Links with local (private) schools? Nos domestic violence cases?

Anything they are particularly proud of? (ie recording policy, contacts, team work, training).

Any difficulties (ie safeguarding lead or previous training)?

**Delivery**

Are issues raised that are not addressed?

Do trainers continue to relate domestic violence and abuse (DVA) and child safeguarding (CS)?

Are participants invited to reflect on and share experiences?

Timing: rushed/OK?

Manual followed? Which parts?

What works well/could be improved?

**Participation/Group**

Do they all (gender/age/role) participate?

Are they willing to reflect and share?

Any participants silent? Excluded? Ignored?

Is there debate/clarification by participants?

What works well/could be improved?

**Engagement with materials/trainers**

How do they respond to the video? Slides? Information?

Do they make or resist links between DVA and CS?

Do they trust/dismiss the materials?

Do they trust trainers? Differences between social worker/health care professional?

What materials/who triggers debate?

Any redundant information (know/in place already)?

Comments about quality of materials/trainers

What works well/could be improved?

**Content/message**

Do they get stuck in particular issues?

Do they trust/resist the message? Believe you can talk to children?

Is there sufficient local content to meet demand?

Is knowledge shared within the practice?

What works well/could be improved?

**Evidence of shifts in perspective or learning in the group**
