# Supplementary material for: Training on domestic violence and child safeguarding in general practice: a mixed method evaluation of a pilot intervention
Source: BMC Fam Pract. 2017 Mar 4;18:33. doi: 10.1186/s12875-017-0603-7 (PMC5336644; doi:10.1186/s12875-017-0603-7)
Supplement: Additional file 3: — Training evaluation interview schedule – trainers. (DOCX 17 kb) [file 12875_2017_603_MOESM3_ESM.docx]

**Consent.** Thanks for agreeing to take part in this interview. We would like to record the conversation. It will be transcribed and then the research team will have access to the transcript, but it won’t be shared with anyone else outside the team. We will use what you say to help us learn about how to deliver and improve the training. We may write about this in reports and journal articles and we may want to use some quotes from what you say, but in a way that would not identify them. Is that all OK?

Switch on recorder…today’s date is…. Your name is … And do I have your consent to record this?

**1. Context**

Can you tell me about the training you delivered to refresh our memories?

What were the practices?

What were the differences between them?

Was there anything particularly difficult or pleasantly surprising about any of them?

Well let’s look at some of that in a bit more depth and I’ll ask you some questions about the training materials, the group dynamics, your role as facilitators and what you think they learned […add anything additional they mentioned as a specific issue].

**2. The materials**

What did you think of the training materials?

Did anything work particularly well?

Did anything not work?

What bits did you leave out? Why? Would you leave out the same bits next time?

Is there anything else that you think should have been included?

What parts of the training materials do you think went down well with the training participants?

Do you think there was the right sort of balance between discussing general principles and giving local multi-agency information?

**3. Delivery and dynamics**

How do you feel about how your team delivered the training?

What were the difficulties?

Timing? Space? Audio video equipment?

Who do you feel you worked as a team?

Teamwork, division of labour, tensions?

Did you find it was possible to get the trainees to share their experiences and ideas?

(Prompt: any reflect on their practice? Can you give me any examples to illustrate that?)

Did everyone participate? Did some people dominate discussions?

(Prompt: who)

Do you think they found it worthwhile?

(Prompt: differences between practices)

Do you think they trusted the training materials?

Do you think they trusted you as trainers?

Did there need to be two people delivering the training?

Is there anything that would make it easier to facilitate the training?

**4. Content/message/learning**

What do you think they learned from the training?

(Prompt: attitudes towards domestic violence and abuse (DVA), child safeguarding (CS) and connections

What to do and confidence to do it

How they see their role

Knowledge and understanding of other agencies' roles and procedures

Knowledge of internal (practice) policy, procedure and expectations)

Can you give me any examples to illustrate that?

Did any people or practices resist the message?

Do you think both nurses and general practitioners benefited equally from this training in general?

If not, why not, and how could this be improved?

Which practices benefited most from this training in your experience?

(Prompt: engaging ones or those who had a generally low baseline, etc)

What else worked well/could be improved?

**5. Finishing Off**

Did the training event for trainers adequately prepare you for delivering the training?

Prompt: what was good? Missing?

What would you advise someone else who was delivering the training?

Any other comments?

Thanks
