# Supplementary material for: Training on domestic violence and child safeguarding in general practice: a mixed method evaluation of a pilot intervention
Source: BMC Fam Pract. 2017 Mar 4;18:33. doi: 10.1186/s12875-017-0603-7 (PMC5336644; doi:10.1186/s12875-017-0603-7)
Supplement: Additional file 4: — Training evaluation interview schedule – training participants. (DOCX 17 kb) [file 12875_2017_603_MOESM4_ESM.docx]

**Consent.** Thanks for agreeing to take part in this interview. We would like to record the conversation. It will be transcribed and then the research team will have access to the transcript, but it won’t be shared with anyone else outside the team. We will use what you say to help us learn about how to deliver and improve the training. We may write about this in reports and journal articles and we may want to use some quotes from what you say, but in a way that would not identify them. Is that all OK?

Switch on recorder…today’s date is…. Your name is … And do I have your consent to record this?

**1. Context**

Can you tell me about what you remember from the training?

Who was there? What room were you in?

What did you learn from the training?

What were the key messages?

Was there anything particularly difficult or pleasantly surprising about it?

Well let’s look at some of that in a bit more depth and I’ll ask you some questions about the training content and how it was delivered and then look at whether you think it has had any impact on your practice.

**2. The materials and delivery**

What did you think of the training content?

What information was useful?

What did you think of how it was delivered?

What did you think of the video?

(Prompt: realistic, emotionally engaging, length, class, ethnicity)

Did anything work particularly well? What was the best bit?

Did anything not work? What was the worst part?

Do you think there was enough opportunity to reflect on your own experience or share cases with colleagues?

Did everyone in the training group take part? If not, how could this be improved?

The training was delivered by a multi-agency team (remind that one was a social worker if necessary) - what would you say were the benefits and drawbacks of this?

(Prompt: what did you think of the social worker input?)

**3. Impact/learning**

Prior to the training, how would you rate you have rated your confidence in dealing with children who have experienced domestic violence and abuse (DVA)? (0-10)

How would you rate this now?

What has changed?

Prior to the training, how would you rate you have your knowledge and skills in dealing with children who have experienced DVA? (0-10)

How would you rate this now?

What has changed?

Has your attitude towards working with domestic violence victims, especially children, altered in any way?

When a couple is in conflict – would you get involved?

If so, when and how?

What level of suspicion would you need in order to ask an adult victim whether she is experiencing DVA?

Have you had any relevant cases since the training? How did/would you proceed?

Has the training changed your thinking or practice in any way – please give me an example

When a child is living in a household where domestic violence is occurring – would you get involved?

If so, when and how?

Have you talked to a child since you had the training? How did/would you proceed?

Has the training changed your thinking or practice in any way – please give me an example

Do you feel you have any more strategies for responding to victims of DVA than you had before the training?

What sorts of activities might fit within your role?

(Prompt:

- getting information,
- providing support,
- consulting with others, a
- accessing specialist services,
- monitoring the situation,
- referring to safeguarding,
- obtaining feedback about other agency involvement)

Has the training had any impact on how you and your colleagues record domestic violence?

Has the training had any impact on your work with other agencies?

(Prompt: awareness of DVA agencies, understanding of social services responses, etc)

**4. Finishing off**

If you were designing this training what would you change?

Any other comments?

Thanks
